# Occupational risks in a representative sample of Portuguese tattoo artists: comparison between self-perception and risk assessment methods

**DOI:** 10.47626/1679-4435-2022-956

**Published:** 2024-08-05

**Authors:** Sandra Mónica Santos

**Affiliations:** 1 Faculdade de Engenharia, Universidade do Porto, Porto, Portugal

**Keywords:** occupational health services, occupational health program, risk factors, occupational medicine, tattooing, risk assessment, serviços de saúde do trabalhador, programa de saúde ocupacional, fatores de risco, medicina do trabalho, tatuagem, avaliação de risco

## Abstract

**Introduction:**

In recent decades there has been a growth in the demand for tattoos and in the number of tattoo artists.

**Objectives:**

A study was designed to compare the results obtained in the application of risk assessment instruments by the Occupational Safety and Health team with those of the risk perception of the same variable in a sample of tattoo artists.

**Methods:**

A risk chart was prepared and the William Fine method, the integrated risk assessment approach, and the methodology for risk assessment and accidents at work were applied for general risk assessment, whereas the Ovako Working Posture Analysis System and Rapid Entire Body Assessment were applied for ergonomic risk assessment. Tattoo artists’ perception was registered in an online questionnaire.

**Results:**

The most valued risk factors by tattoo artists were forced/maintained postures and repetitive movements; conversely, interaction with old machines and/or in poor condition and monotonous work. Divergences were found when comparing the results of risk assessment with those of risk perception, since the first highlights chemical and biological agents. This may be justified by the fact that tattoo artists give more relevance to issues capable of causing faster and/or more intense semiology.

**Conclusions:**

If the Occupational Safety and Health team is attentive and prepared to deal with these differences, it will achieve better performance.

## Abstract (pt)

**Introdução:**

Nas últimas décadas, houve um crescimento na procura por tatuagens e no número de tatuadores.

**Objetivos:**

Projetou-se um estudo para comparar os resultados obtidos na aplicação de instrumentos para avaliação de risco pela equipe de Saúde e Segurança Ocupacionais com a percepção da mesma variável em uma amostra de tatuadores.

**Métodos:**

Elaborou-se uma carta de riscos, e foram aplicados os métodos de William Fine, a metodologia integrada de avaliação de risco, bem como e de avaliação do risco e acidentes de trabalho para risco geral e o Ovako Working Posture Analysis System e Rapid Entire Body Assessment para o risco ergonômico. A percepção dos tatuadores foi registrada em um questionário *on-line*.

**Resultados:**

Os fatores de risco mais valorizados pelos tatuadores foram as posturas forçadas/mantidas e os movimentos repetitivos; por sua vez, entre os fatores de risco menos valorizados, estavam a interação com máquinas antigas e/ou em mau estado e o trabalho monótono. Comparando os resultados da avaliação de risco com a percepção de risco, constata-se que existem divergências, uma vez que a primeira realça os agentes químicos e biológicos. Isso pode se justificar por eventualmente os tatuadores darem mais relevância às questões com capacidade para originar semiologia mais rápida e/ou intensa.

**Conclusões:**

Se a equipe de Saúde e Segurança Ocupacionais estiver atenta e preparada para lidar com essas diferenças, terá maior capacidade de obter melhor desempenho.

## INTRODUCTION

Tattooing may be defined as the introduction of pigment into the dermis, desirably in a permanent manner, using a needle.^1-3^ In the last decades, a significant growth has been observed in the demand for tattoos worldwide, stimulating the supply, as confirmed by the increase in the number of facilities and professionals in this area. However, this evolution was not accompanied by support from the area of Occupational Safety and Health, which continues to provide tattoo artists with standard services, little adapted to their characteristics, thus possibly leading to the perception that these services have little utility, which makes that they are not always used,^1^ despite knowing about their legal obligation.

Tattoo artists are exposed to several occupational risk factors, in which they need to have training. Examples of these factors include the several chemical agents that tattooers handle (especially pigment components and hygiene products),^2-6^ which may cause dermatological, otorhinolaryngological, and even oncological changes^2,4,7,8^; and the biological agents possibly arising from contact with client’s blood (through cutaneous/ mucosal/percutaneous routes), which may have infections by the human immunodeficiency virus (HIV) and/or hepatitis (B and C) as a consequence.^1,2,4,8-10^

Forced/maintained postures resulting from prolonged shifts (in situations of large tattoos and/or with several appointments in a row, without intervals) and the performance of repetitive movements (tattooing and cleaning the skin from excess pigment, a number of times subsequently) may give rise to orthopedic diseases, due to muscle, tendon, and/or joint wearing out. Other risk factors is working in a poorly illuminated environment, which may lead to visual fatigue and/or reduced visual acuity, due to visual demand; handling of electric tattooing machines for long periods, with continuous exposure to noise^4,9^ (nearly 15 decibels) and to low intensity vibrations,^4,9^ which may be associated with hearing loss and vascular/neurological disturbances, respectively; as well as with exposure to psychosocial factors, with stress standing out (associated with management of tattoo appointments, with an empty schedule, or with guidance on clients’ expectations about the final product presented, which is potentially irreversible).

Risk perception, in turn, describes how an individual perceives susceptibility to a specific damage, secondary to a threatening event, and depends on beliefs, attitudes, values, and personality; therefore, it is subjective.^11^ Risk assessment, conversely, is more objective. Taking this difference into account, the results for perception are usually different from those of the risk assessment.^12^

### OBJECTIVES

Considering these premises and the scarce scientific literature, both on the objective assessment of occupational risks present in the sector and on subjective risk perception by tattooists, an exploratory study was designed aiming to compare the results obtained in the application of certified risk assessment instruments, namely the William Fine method, the integrated risk assessment (MIAR) approach, and the methodology for risk assessment and accidents at work (metodologia de avaliação do risco e acidentes de trabalho, MARAT), with workers’ self-perception from a representative sample of professional Portuguese tattoo artists, by means of an online survey.

Therefore, by comparing the differences between a more objective professional assessment and a more subjective personal assessment, the Occupational Safety and Health team will be more able to obtain better performance, since they will understand more clearly how to approach, disseminate, train, and guide.

## METHODS

Initially, a generic risk chart was created covering the tasks usually performed by tattoo artists during professional practice. Subsequently, the William Fine method, the MIAR approach, and the MARAT were applied to assess general risk, and the Ovako Working Posture Analysis System (OWAS) and the Rapid Entire Body Assessment (REBA) for ergonomic risk assessment in particular.

To measure tattoo artists’ perception on the different occupational risk factors, an online questionnaire was created and made available from April 2020 to March 2021, using the Google Forms platform. It was directly disseminated in tattooing companies/professionals, national magazines of the sector, companies that supply products and equipment, and organizing committees of national tattooing congresses Based on the number of professionals registered in the Portuguese tax office, this study sought to obtain a representative sample of the study population, considering a sampling error of 5% and a confidence interval (CI) of 90%.

With regard to statistical analysis, after checking for the normality of the variables with the ShapiroWilk test, the chi-square and the Kruskal-Wallis tests were predominantly used to investigate the differences between the variables, as well as the Spearman’s correlation coefficient.

The investigation project was approved by the Research Ethics Committee of Faculdade de Letras da Universidade do Porto, and all participants provided free informed consent.

## RESULTS AND DISCUSSION

A total of 207 tattoo artists answered the questionnaire, which accounts for 25.9% of the professionals registered in Portugal.

### GENERAL RISK ASSESSMENT

Although the three methods used (William Fine method, MIAR and MARAT) value different aspects, it was interesting to observe the homogeneity of results. The highest risk category includes possible contacts with chemical agents, sharp objects, and potentially contaminated blood, as well as cleaning of skin (of the excess pigment).

Although the MIAR approach indicated two items that the other techniques did not include in this category (contact with blood during cleaning/disinfection/ sterilization of working surfaces and instruments). The method that most valued the occupational risk factors in general was the MIAR approach (9 items in the highest risk category and 37 in the lowest risk category), the opposite being the William Fine method (7 and 46, respectively).^13^

### ERGONOMIC RISK ASSESSMENT

According to the OWAS method, it was observed that the tasks of making the drawing on paper or searching from a design on the computer and inserting it on decal sheets obtained the lowest risk level(1); all the other tasks were classified into the action category 2, except for the possibility of the tattoo artist having to help the client, which obtained a rating of 4, in view of its load bearing. However, when weighting the time that each task usually occupies in percentage in relation to the work shift, the situation changed, i.e., the action level 4 disappeared, because tattoos artists would be able, in a few seconds, to put themselves and the client in a less forced posture.

Moreover, with this percentage adjustment, nearly half of action levels 2 went to 1, with the tasks of drawing the pattern that will be tattooed directly on the skin, injecting pigment into the skin, cleaning the excess pigment, and applying other chemical agents to the epidermis remaining in the first value, as well as cleaning/disinfection/sterilization of working surfaces and instruments (also including their accommodation).^14^

In turn, in the REBA methodology, none of the tasks considered had action level 0, and only one had the highest level, i.e., level 4 (helping the client). At action level 2, the tasks considered were shaving, disinfecting/ sterilizing the skin, transferring the drawing from the decal sheet to client’s skin, putting chemical agents other than pigment on the skin during tattooing, and cleaning/disinfecting/sterilizing work surfaces. At action level 3, there remained the tasks of drawing the pattern that will be tattooed directly on the skin, inserting the drawings on the decal sheets, preparing the workbench/ work tray, injecting the pigment on the skin, cleaning the skin of the excess pigment, and cleaning/disinfecting/ sterilizing/accommodating the work instruments.^14^

It was found that the OWAS methodology was able to better value the risk, considering the time that the task occupies, when compared to the REBA technique, although the probability of the event occurring or not is not considered in any of the methodologies.^14^

Overall, the different results would eventually be attenuated if the tasks were divided into subtasks; however, this would increase the complexity of the assessments, even in low-diversified jobs. Furthermore, other condition may have biased the results: or only and exactly a single moment is assessed with rigor (running the risk of biasing whether professionals have positioned themselves correctly or not and not considering the risk that other postures would bring the same task, in other moments of tattooing and/or even with other tattoo artists), or, in an attempt to prevent against such, consider the most serious possibility. Since the objective was to portray the tattoo sector globally (and not one studio or tattoo artist in particular), it was decided to cover all situations and, when there are several possible ones, always consider the most serious.^14^

Moreover, even though these methods allow some subjectivity to be mitigated in the risk assessment, it cannot be eliminated. In situations of doubt between two hypotheses of the scale, an evaluator may choose one hypothesis at one time and, in another equivalent task, choose the other. This can even happen when repeating the evaluation of exactly the same task at different times. Even so, these methods constitute a valuable aid in risk assessment.^14^

### SELF-PERCEPTION TOWARDS OCCUPATIONAL RISKS

With regard to the characterization of the sample of tattoo artists assessed, age ranged from 20 to 52 years, with a median of 34 years and a mode of 36 years. In terms of sex, as described in the scientific literature, there was predominance of the male sex (66.7%). In relation to **Table 1.** Valuation of occupational risks by tattoo artists schooling, elementary (10.6%) and high school (59.4%) education still prevail, although higher education accounted for 30%. Even though all participants worked in Portugal and mastered the Portuguese language, 8.2% did not have Portuguese nationality.

Professionally, the tattoo artists assessed were very heterogeneous, including workers with little professional experience (less than 1 year) and other very experienced ones (more than 30 years), with a median of 5 years. Exclusive dedication to tattooing prevails (60.9%), but only 58% of individuals received previous training to start working professionally, ranging from those who attended short courses, who did an internship, and who worked as trainees of other tattoo artists.

When participants were asked whether they considered to be exposed to occupational risks, they were unanimous in acknowledging the presence of these risks. However, when they were asked to classify exposure to risk based on an exhaustive list of potential risk factors related to professional practice, it was observed that, although some of them stood out, all risk factors were assigned with some risk classification (Table 1).

**Table 1:** Valuation of occupational risks by tattoo artists

	Null (%)	Low (%)	Medium (%)	High (%)
Chemical agents	9.9	38.4	37.9	13.8
Forced/maintained postures	0.5	2.9	21.1	75.5
Repetitive movements	0.5	8.9	27.1	63.5
Machines capable of causing injuries	9.9	40.9	30.0	19.2
Old machines and/or in poor condition	60.9	19.8	11.9	7.4
Noise	22.7	52.7	19.2	5.4
Vibrations	10.1	43.9	31.8	14.1
Eyestrain, inadeguate lighting	12.8	24.1	34.0	29.1
Biological agents	10.4	19.9	30.3	39.3
Isolated work	28.4	38.3	21.9	11.4
Monotonous work	55.7	31.5	9.9	3.0
Prolonged shifts	19.3	24.8	36.6	19.3
Stress	21.4	33.3	30.3	14.9

The most valued risk factors were forced/maintained postures and repetitive movements; in an opposite situation there were interaction with old machines and/ or in poor condition and monotonous work.

When associating the two highest levels of risk perception (medium and high), forced/maintained postures (96.6%) and repetitive movements (90.6%) continue standing out. These factors usually cause symptoms such as musculoskeletal pain and, thus, are more present in tattoo artists’ perception, especially when working for long periods (55.9%).

Despite being moderately present, biological risk resulting from handling of blood (69.6%) have a considerably lower percentage than that of the most mentioned factors on Table 1, which may be associated with a valuation of the subject that led tattoo artists to previously take the required precaution measures, thus not emphasizing the potential current risk, by comparison with other risk factors that were left unnoticed at an initial stage and currently are perceived as more intense.

**Figure 1:**
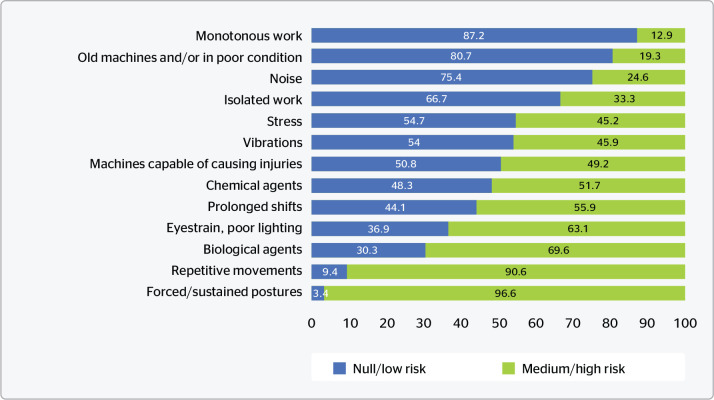
Percent distribution of perception of severity of exposure to occupational risk factors.

The next risk factor is eyestrain (63.1%), resulting from handling the tattooing machine often without the required lighting and for long periods. Finally, but still with a percentage above 50%, there is handling of chemical agents (51.7%), associated both with the inks and with cleaning of skin, work instruments, and work surfaces (Figure 1).

In the opposite direction, associating the two lowest levels of risk perception (null and low), there are, in an ascending order, monotony associated with the tasks (12.9%); handling of tattoo machines in poor state of conservation (19.3%); and exposure to noise (24.6%) which is of low intensity and thus is not usually interpreted as a risk factor that requires relevant measures to be taken (Figure 1).

Moreover, it is possible that, generically, some workers give a greater value to risk factors with more immediate and/or intense consequences and/or symptoms rather than other factors that (despite leading to significant situations) do not lead to significant symptoms or relevant interaction with quality of life at that time.

### RELATIONSHIP OF SOCIODEMOGRAPHIC AND PROFESSIONAL VARIABLES WITH RISK PERCEPTION

Results reveal that the sex is important in risk perception, since the female sex usually perceives risk more intensely, as described in the literature,^12,15,16^ which was more noticeable in relation to chemical agents (p = 0.035), forced/maintained postures (p = 0.008), and to stress (p = 0.007).

With regard to age, it was observed that older age groups have a more intense perception of exposure to occupational risk, emphasizing the performance of repetitive movements (p = 0.013), which are more likely to cause painful symptoms in older individuals (with lower functional capacity), and handling of chemical agents (p = 0.019).

An analysis of professional experience revealed that professionals who work for a longer time in the sector usually classify risk as more intense, with a statistically significant difference for repetitive movements (p = 0.005), noise (p = 0.036), and eyestrain (p = 0.018), which may explained by the probable presence of symptoms such as musculoskeletal pain, visual fatigue, and reduced auditory acuity in individuals whose period of exposure was longer.

Simultaneously, professionals who work exclusively in the sector of tattooing perceive the several risk factors more intensely, perhaps because they are more exposed, especially the performance of repetitive movements (p = 0.010) and exposure to stress (p = 0.050), in which differences are statistically significant. Professionals who work simultaneously in another job are more likely to devalue some factors, such as vibrations (p = 0.003), which were classified as null risk by most professionals, a finding that may be justified by their shorter exposure to hazard.

The presence of correlations between tattoo artists’ perception towards the different risk factors was investigated using Spearman’s correlation coefficient (Table 2).

**Table 2:** Statistical correlations between tattoo artists’ perception towards different occupational risk factors

	Chemical products	Forced/ maintained postures	Repetitive movements	Machines capable of causing lesions	Machines in poor condition	Noise	Vibrations	Eyestrain/ poor lighting	Biological agents	Isolated work	Monotonous work	Prolonged shift	Stress
Chemical products Coef.	1.000	0.123	0.051	0.194+	0.358+	0.160^*^	0.117	0.232+	0.313+	0.107	0.124	0.116	0.171^*^
Significance		0.080	0.474	0.006	0.000	0.023	0.100	0.001	0.000	0.132	0.079	0.101	0015
n	203	203	202	203	202	203	198	202	201	200	203	202	201
Forced/maintained postures Coef.	0.123	1.000	0.533†	0.200+	0.112	0.072	0.169^*^	0.221+	0.181+	-0.003	-0.037	0.145^*^	0.203+
Significance	0.080		0.000	0.004	0.113	0.306	0017	0.002	0.010	0.969	0.596	0.040	0.004
n	203	204	203	203	202	203	198	203	201	200	203	202	201
Repetitive movements Coef.	0.051	0.533+	1.000	0.244+	0.114	0.143^*^	0.289+	0.306+	0.251+	0.104	0.156^*^	0.243+	0.294+
Significance	0.474	0.000		0.000	0.108	0.042	0.000	0.000	0.000	0.145	0.027	0.001	0.000
n	202	203	203	202	201	202	197	202	200	199	202	201	200
Machines capable of causing injuries Coef.	0.194^†^	0.200^†^	0.244+	1.000	0.435+	0.364+	0.347+	0.349+	0.390+	0.092	0.165^*^	0.153^*^	0.182+
Significance	0.006	0.004	0.000		0.000	0.000	0.000	0.000	0.000	0.195	0019	0.030	0.010
n	203	203	202	203	202	203	198	202	201	200	203	202	201
Machines in poor condition Coef.	0.358+	0.112	0.114	0.435+	1.000	0415+	0.354+	0.466+	0.397+	0.227+	0.204+	0.248+	0.236+
Significance	0.000	0.113	0.108	0.000		0.000	0.000	0.000	0.000	0.001	0.004	0.000	0.001
n	202	202	201	202	202	202	197	201	200	199	202	201	200
Noise Coef.	0.160^*^	0.072	0.143^*^	0.364+	0.415+	1.000	0.589+	0.363+	0.284+	0.247+	0.186+	0.299+	0.316+
Significance	0.023	0.306	0.042	0.000	0.000		0.000	0.000	0.000	0.000	0.008	0.000	0.000
n	203	203	202	203	202	203	198	202	201	200	203	202	201
Vibrations Coef.	0.117	0.169^*^	0.289^†^	0.347+	0.354+	0.589+	1.000	0.444+	0.300+	0.200+	0.151^*^	0.257+	0.244+
Significance	0.100	0017	0.000	0.000	0.000	0.000		0.000	0.000	0.005	0.033	0.000	0.001
n	198	198	197	198	197	198	198	197	196	195	198	197	196
Eyestrain/poor lighting Coef.	0.232+	0.221+	0.306+	0.349+	0.466+	0.363+	0.444+	1.000	0.524+	0.124	0.119	0.297+	0.278+
Significance	0.001	0.002	0.000	0.000	0.000	0.000	0.000		0.000	0.081	0.091	0.000	0.000
n	202	203	202	202	201	202	197	203	200	199	202	201	200
Biological agents Coef.	0.313+	0.181^†^	0.251^†^	0.390+	0.397^†^	0.284+	0.300+	0.524+	1.000	0.166^*^	0.153^*^	0.312+	0.237+
Significance	0.000	0.010	0.000	0.000	0.000	0.000	0.000	0.000		0.019	0.030	0.000	0.001
n	201	201	200	201	200	201	196	200	201	198	201	200	199
Isolated work Coef.	0.107	-0.003	0.104	0.092	0.227+	0.247+	0.200+	0.124	0.166^*^	1.000	0.446+	0317+	0.205+
Significance	0.132	0.969	0.145	0.195	0.001	0.000	0.005	0.081	0.019		0.000	0.000	0.004
n	200	200	199	200	199	200	195	199	198	201	200	199	198
Monotonous work Coef.	0.124	-0.037	0.156^*^	0.165^*^	0.204+	0.186+	0.151^*^	0.119	0.153^*^	0.446+	1.000	0.189+	0.305+
Significance	0.079	0.596	0.027	0.019	0.004	0.008	0.033	0.091	0.030	0.000		0.007	0.000
n	203	203	202	203	202	203	198	202	201	200	203	202	201
Prolonged shifts Coef.	0.116	0.145^*^	0.243+	0.153^*^	0.248^†^	0.299+	0.257+	0.297+	0.312+	0.317+	0.189+	1.000	0.381+
Significance	0.101	0.040	0.001	0.030	0.000	0.000	0.000	0.000	0.000	0.000	0.007		0.000
n	202	202	201	202	201	202	197	201	200	199	202	202	201
Stress Coef.	0.171^*^	0.203+	0.294+	0.182+	0.236+	0.316+	0.244+	0.278+	0.237+	0.205+	0.305+	0.381+	1.000
Significance	0.015	0.004	0.000	0.010	0.001	0.000	0.001	0.000	0.001	0.004	0.000	0.000	
n	201	201	200	201	200	201	196	200	199	198	201	201	201

* Correlation is significant at the 0.05 level (2 ends); † Correlation is significant at the 0.01 level (2-tailed). Coef. = Spearman’s correlation coefficient.

Professionals who highlighted forced/maintained postures were more likely to value repetitive movements (rho = 0.053; p < 0.001), prolonged shifts (rho = 0.145; p = 0.004), and stress (rho = 0.203; p = 0.004). Likewise, those who most value repetitive movements also mention monotonous work (rho = 0.157; p = 0.027), prolonged shifts (Rho = 0.243; p < 0.001), and stress (rho = 0.294; p < 0.001). Finally, it bears noting the positive correlation between the intensity with which tattoo artists perceive prolonged shifts and stress (rho = 0.381; p < 0.001).

An assessment of the intensity of perception towards eyestrain showed that it is positively correlated with the valuation attributed to prolonged shifts (rho = 0.297; p < 0.001), repetitive movements (rho = 0.306; p < 0.001), and stress (rho = 0.278; p < 0.001).

In turn, valuation of noise as an occupational risk factor was positively correlated with risk perception associated with machines capable of causing injuries (rho = 0.364; p < 0.001), instruments in poor condition (rho = 0.415; p < 0.001), vibrations (rho = 0.589; p < 0.001), prolonged shifts (rho = 0.299; p < 0.001), and stress (rho = 0.316; p < 0.001).

It was also found that valuation of isolated work as a risk factor was positively and moderately correlated with monotonous work (rho = 0.446; p = 0.001) and stress (rho = 0.205; p = 0.004), whereas monotonous work was correlated with prolonged shifts (rho = 0.189; p = 0.007) and stress (rho = 0.300; p < 0.001).

Finaly, perception of chemical risk was found to be positively and moderately correlated with biological risk (rho = 0.313; p < 0.001).

## CONCLUSIONS

The job of tattoo artist is still little regulated and standardized, being accessible to any person who wants to start working as a self-employed or at some preexisting studio, taking into account that training addressing risk factors associated with professional practice is scarce or reduced. Simultaneously, it is also a labor area still little or not investigated in the context of Occupational Safety and Health. This exploratory study, in addition to making it possible to assess the risk perceptions expressed by Portuguese tattoo artists, allowed for building a chart of risks, with the application of different previously certified instruments.

For risk assessment (global or ergonomic), it is recommended to use methodologies that enable develop a mathematical hierarchy of the list of problems, guiding tattoo artists with regard to the required changes to be done, both from the structural point of view and in procedures. However, comparing the results for risk assessment with those for risk perception, it is observed that results are divergent, i.e., the first emphasizes chemical and biological agents, whereas the latter highlights forced/maintained postures and repetitive movements.

Biological agents, from the professionals’ point of view, seem to be less valued (comparatively), but, even so, are more emphasized than other risk factors. However, this risk has the potential of causing irreversible damage, being thus necessary to develop techniques that increase safety and effectively protect workers’ health, in addition to promoting appropriate professional training. Nonetheless, this alleged lower valuation may be justified by the fact that, in the past, biological agents were one of the most highlighted risk factors (comparatively to the others) and that this could have provided an appropriate approach, so that tattoo artists consider that the actual risk is lower, given the protective measures currently implemented in all studios.

This may be justified by the fact that tattoo artists eventually give more relevance to issues capable of causing faster and/or more intense semiology, compared with risks that have later and/or milder consequences. If the Occupational Safety and Health team is attentive and prepared to deal with these differences, they will be more able to achieve better performance.

## References

[1] Cortelli AFD. (2012). Procedimentos de biossegurança adotados por profissionais
prestadores de serviços de manicure, pedicure, tatuagem, piercing e
maquiagem definitiva no município de Jacareí - SP
[Dissertação de Mestrado].

[2] Ramos BAO. (2018). Desenvolvimento de métodos eletroquímicos para
análise de agentes tóxicos em tintas de tatuagem.

[3] Serup J, Linnet J, Olsen O, Harrit N, Mohl B, Westh H. (2015). Tattoos - Health, risks and culture.

[4] Kluger N. (2017). National survey of health in the tattoo industry: observational
study of French Tattooists. Int J Occup Med Environ Health.

[5] Council of Europe, Committee of Ministers. Resolution ResAP (2003). on tattoos and permanent make-up.

[6] Campos S, Lestre S, João A, Lobo L. (2013). Exantema mercurial com reação pustularuma forma de
dermatite de contato sistémica associada à tatuagem.

[7] Kluger N. (2015). Pregnancies in tattooed female tattooists: an observational
study. Eur J Obst Gynecol Reproductive Biol.

[8] Piccinini P, Pakalin S, Contor L, Bianchi I. (2016). Safety of tattoos and permanent make-up: adverse health effects and
experience with the Council of Europe Resolution (2008)1.

[9] Grieshaber DC, Marshall MM, Fuller T. (2012). Symptoms of musculoskeletal disorders among tattoo
artists. Proc Hum Factors Ergon Soc Annu Meet.

[10] Molina L, Romiti R. (2011). Molusco contagioso em tatuagem. An Bras Dermatol.

[11] Alexandre P. (2017). Exposição ocupacional a agentes antineoplásicos -
Percepção de risco [Dissertação de
Mestrado].

[12] Jin J, Wang W, He R, Gong H. (2017). Pesticide use and risk perceptions among small-scale farmers in
Angiu Country, China. Int J Environ Res Public Health.

[13] Santos M. (2020). Avaliação de riscos no setor da tatuagem: podem
utilizar-se os métodos MARAT, William Fine e MIAR?. Rev Port Saude Ocup Online.

[14] Santos M. (2020). Avaliação ergonómica das tarefas executadas
no setor da tatuagem: podem usar-se os métodos OWAS e
REBA?. Rev Port Saude Ocup Online.

[15] Sun C, Ahn C, Yang K, Stentz T, Kim H., Duffy V (2017). Digital human modeling. Applications in health, safety, ergonomics, and
risk management: Health and safety.

[16] Kao K, Spitzmuleller C, Cigularov K, Thomas C. (2019). Linking safety knowledge to safety behaviors: a moderated
mediation of supervisor and work safety attitudes. Eur J Work Organiz Psychol.

